# Options for the recovery of mental activity in children after acute brain damage

**DOI:** 10.1192/j.eurpsy.2021.658

**Published:** 2021-08-13

**Authors:** Y. Sidneva, A. Zakrepina, M. Bratkova, S. Valiullina

**Affiliations:** 1 The Department Of Rehabilitation; Psychiatric Research Group, Clinical and Research Institute of Emergency Pediatric Surgery and Trauma; N.N.Burdenko National Medical Research Center of Neurosurgery, Moscow, Russian Federation; 2 The Department Of Rehabilitation; Laboratory Of Psychological And Pedagogical Research And Technologies For Special Education Of Persons With Intellectual Disabilities, Clinical and Research Institute of Emergency Pediatric Surgery and Trauma; The Federal State Budget Scientific Institution “Institute of Special Education of the Russian Academy of Education”, Moscow, Russian Federation; 3 Institute Of Special Education And Psychology Institute Of System Projects Institute Of Lifelong Learning Directorate Of Educational Programs Institute Of Education Content, Methods And Technology, Moscow City University; Clinical and Research Institute of Emergency Pediatric Surgery and Trauma, Moscow, Russian Federation; 4 Department Of Rehabilitation, Clinical and Research Institute of Emergency Pediatric Surgery and Trauma (CRIEPST), Moscow, Russian Federation

**Keywords:** recovery of mental activity, rehabilitation of children, minimal consciousness, vegetative status

## Abstract

**Introduction:**

Children with acute brain damage make up a large group of patients who require multi-stage rehabilitation. Rehabilitation requires the creation of special conditions for psychiatric care and psychological and pedagogical correction of the consequences of severe damage to the nervous system.

**Objectives:**

To identify the options for mental activity during the restoration of the level of consciousness in children after acute severe brain damage.

**Methods:**

210 children under the age of 18 with severe brain damage (traumatic brain injury, hypoxia, hydrocephalus). Clinical-psychopathological, pedagogical methods were used; additionally diagnostic scales, questionnaires.

**Results:**

4 groups were formed: 1st 37 (18%) patients had manifestations of mental activity with physical, cognitive and social capabilities in the minimal consciousness “+” (a- / hyperkinetic mutism with emotional reactions, understanding of addressed speech); 2nd 67 (32%) - manifestations of physical and cognitive abilities with minimal consciousness “-” (a- / hyperkinetic mutism without reactions); 3rd 95 (40%) - only the manifestation of physical capabilities at the exit from the vegetative status. 4th 11 (10%) - a low manifestation of mental activity in the form of physical capabilities with a vegetative status.

**Conclusions:**

4 variants of mental activity in children after acute severe brain damage have been identified: from minimal involuntary reactions or their absence in vegetative status to voluntary actions according to the instructions of an adult in minimal consciousness “+”. Taking into account the variability of mental activity helps to differentiate the methods of psychiatric and psychological-pedagogical assistance in the recovery of children already in the early stages of rehabilitation.

